# Supplementary material for: Staphylococcus aureus Infection-Related Glomerulonephritis with Dominant IgA Deposition
Source: Int J Mol Sci. 2022 Jul 5;23(13):7482. doi: 10.3390/ijms23137482 (PMC9267153; doi:10.3390/ijms23137482)
Supplement: Supplementary file 1 [file ijms-23-07482-s001.zip › Table S1.pdf]

Table S1. Case reports of IgA-IRGN or SAGN.

Ref. No.	Authors	Year	category: IgA-IRGN	category: SAGN	age	gender	underlying diseases: diabetes	underlying diseases: cancer	Causative bacteria	Infection sites
14	Garcia RA, et al.	2021	Yes	Yes	70	M	no	no	S. aureus	LVAD
15	Nogueira RF, et al.	2021	Yes	Yes	66	M	no	no	S. aureus	vascular graft
16	Sahoo RR, et al.	2021	Yes	Yes	40	M	no	no	S. aureus	cellulitis
17	Pérez A, et al.	2021	Yes	No	88	M	no	no	SARS-CoV2	pneumonia
18	Han W, et al.	2021	Yes	No	82	M	no	no	unknown	unknown
19	Hellwege RS, et al.	2020	NA	Yes	23	M	no	no	S. aureus	IE
20	Brunet A, et al.	2020	NA	Yes	27	M	no	no	S. aureus	IE
		2020	Yes	Yes	74	M	no	no	S. aureus	spondylodiscitis & IE
21	Zhang Y, et al.	2020	Yes	Yes	47	M	yes	no	S. aureus	pneumonia
22	Nguyen N, et al.	2020	Yes	Yes	57	M	yes	no	S. aureus	AV graft
23	Indhumathi E, et al.	2020	Yes	Yes	60	M	no	no	S. aureus & M. leprae	skin
24	Shirai Y, et al.	2020	Yes	No	5	M	no	no	parvovirus19	unknown
25	Klimko A, et al.	2020	Yes	Yes	43	M	no	no	Staphylococcus	skin
26	Völker LA, et al.	2019	No	Yes	43	F	no	no	S. epidermidis	VA shunt
27	Carbayo J, et al.	2019	Yes	Yes	70	M	no	no	S. aureus	osteomyelitis
28	AlQahtani H, et al.	2019	Yes	Yes	81	M	no	yes	S. aureus	osteomyelitis
29	Parente YDM, et al.	2019	Yes	No	55	F	no	no	bacilli agglomerates	unknown
30	Molina-Andújar A, et al.	2019	No	Yes	69	M	no	no	S. aureus	unknown
		2019	Yes	No	65	M	no	no	H. zoster / Influenza A	unknown
31	Orozco Guillén AO, et al.	2019	Yes	No	16	F	no	no	syphilis (Treponema)	unknown
32	Cascais de Sá D, et al.	2018	Yes	Yes	56	M	no	no	S. aureus	pyelonephritis
33	Shimamura Y, et al.	2018	Yes	Yes	80	M	yes	no	S. aureus	lung abscess
34	Basic-Jukic N, et al.	2018	Yes	Yes	65	M	yes	no	E. coli	urinary tract
35	Mahmood T, et al.	2018	Yes	Yes	64	M	yes	no	S. aureus	epidural abscess
36	Liang JH, et al.	2016	Yes	Yes	22	F	no	no	S. aureus	skin
37	Okada M, et al.	2016	No	Yes	12	M	no	no	S. epidermidis	CV devise
		2016	No	Yes	24	F	no	no	S. epidermidis	CV devise
38	Srinivasaraghavan R, et al.	2016	Yes	No	12	F	no	no	unknown	unknown
39	Nayer A, et al.	2016	Yes	Yes	82	M	yes	no	S. aureus	osteomyelitis
40	Caetano J, et al.	2015	Yes	Yes	56	M	yes	no	S. aureus	epidural abscess
41	Hayashi S, et al.	2015	No	Yes	45	M	no	no	Staphylococcus	CV devise
42	Rus RR, et al.	2015	Yes	No	3	M	no	no	Streptococcus	rhumatoid fever
43	Saad M, et al.	2015	Yes	Yes	86	M	no	no	S. aureus	pneumonia
44	Kikuchi H, et al.	2014	Yes	No	76	M	yes	yes	E. coli	cholangitis
45	Mandai S, et al.	2013	Yes	Yes	37	M	no	no	S. aureus	Otitis & skin
46	Erqou S, et al.	2012	Yes	Yes	61	M	yes	yes	S. aureus	knee joint
47	Kimata T, et al.	2012	Yes	Yes	6	F	no	no	S. aureus	pneumonia
48	Chen YR, et al.	2011	Yes	Yes	57	M	no	no	S. aureus	hip joint
49	Wehbe E, et al.	2011	Yes	Yes	73	M	yes	yes	S. aureus	pleuritis
		2011	Yes	Yes	69	F	no	no	S. aureus	PM lead infection
50	Kapadia AS, et al.	2011	Yes	Yes	39	M	no	no	S. aureus	psoas abscess
51	Upadhaya BK, et al.	2010	Yes	No	15	M	no	no	dengue virus	unknown
52	Riley AM, et al.	2009	Yes	Yes	64	M	yes	no	S. aureus	osteomyelitis
53	Okuyama S, et al.	2008	Yes	Yes	48	M	no	no	S. aureus	mediastinal abcess
54	Kusaba T, et al.	2008	No	Yes	59	F	no	yes	S. epidermidis	CV devise
55	Ho CI, et al.	2008	No	Yes	70	F	yes	no	S. aureus	osteomyelitis
56	Kimmel M, et al.	2008	NA	No	36	F	no	no	propionibacterium	PM lead infection
57	Hashimoto M, et al.	2007	Yes	Yes	28	F	no	no	S. aureus	genital infection
58	Hoshino C, et al.	2007	NA	Yes	59	F	no	no	S. aureus	pneumonia + abscess
59	Long JA, et al.	2006	Yes	Yes	66	M	no	no	S. aureus	sternoclavicular joint
60	Kitamura T, et al.	2006	Yes	Yes	66	M	no	yes	S. aureus	abdominal abscess
61	Ohara S, et al.	2006	No	Yes	13	M	no	no	S. epidermidis	CV devise
62	Iyoda M, et al.	2006	Yes	No	88	F	no	no	Chlamydia pneumoniae	pneumonia
63	Manzoor K, et al.	2005	No	Yes	40	F	no	no	S. aureus	IE
64	Cheema SR, et al.	2004	Yes	No	61	M	no	no	hepatitis A	liver
65	Handa T, et al.	2003	Yes	Yes	57	F	no	no	S. aureus	pharygeal, stool, skin
66	Arrizabalaga P, et al.	2003	Yes	Yes	72	M	no	yes	Staphylococcus	CV devise
67	Pola E, et al.	2003	Yes	Yes	30	M	no	no	S. aureus	knee joint
68	Peel R, et al.	2003	No	Yes	60	M	no	no	S. aureus	lumber disk
69	Kubota M, et al.	2001	No	Yes	52	M	no	no	S. epidermidis	VA shunt
70	Balogun RA, et al.	2001	No	No	74	M	no	no	propionibacterium	VA shunt & pneumonia
71	Yamashita Y, et al.	2001	No	Yes	58	M	no	yes	S. aureus	pneumonia
72	Barnadas MA, et al.	1998	Yes	Yes	82	M	no	no	S. aureus	skin
73	Griffin MD, et al.	1997	Yes	Yes	72	M	no	no	S. aureus	wound
74	Kitazawa M, et al.	1997	No	No	68	M	yes	no	S. aureus	pneumonia
75	Pulik M, et al.	1995	No	Yes	71	F	no	yes	S. aureus	CV device

IgA-IRGN, IgA-dominant deposition infection-related glomerulonephritis; SAGN, Staphylococcus infection-associated glomerulonephritis; M, male; F, female; LVAD, ; IE, infective endocrditis; AV, arteriovenous; VA, ventriculoatrial; CV, central venous; PM, pacemaker

Ref. No.	symptomes					Laboratory data					
	AKI or RPGN	nephrotic syndrome	proteinuria	hematuria	purpura	proteinuria (g/gCr)	serum creatinine (mg/dL)	serum IgA levels	serum C3 levels	serum C4 levels	positive test for ANCA
14	present	NA	NA	present	present	NA	6.98	NA	NA	NA	NA
15	present	abscent	present	present	abscent	0.6	5.26	NA	normal	normal	postitive
16	abscent	abscent	present	present	abscent	1.4	2.20	NA	decreased	decreased	NA
17	present	present	present	present	abscent	3.8	2.28	NA	normal	decreased	negative
18	present	present	present	present	abscent	4.5	3.12	high	normal	normal	negative
19	present	abscent	present	NA	abscent	10.0	1.60	NA	decreased	normal	negative
20	abscent	abscent	abscent	abscent	present	NA	NA	NA	normal	decreased	negative
	present	present	present	present	abscent	2.4	2.44	NA	normal	normal	negative
21	present	abscent	present	present	abscent	NA	1.76	normal	normal	normal	negative
22	present	present	present	present	abscent	7.2	5.20	NA	decreased	NA	NA
23	present	NA	present	present	present	NA	4.10	NA	decreased	normal	negative
24	present	abscent	present	present	abscent	9.1	1.94	NA	NA	NA	negative
25	present	present	present	present	abscent	3.7	3.22	NA	NA	NA	negative
26	abscent	abscent	present	present	abscent	0.0	2.07	NA	decreased	NA	NA
27	present	present	present	present	abscent	5.4	3.54	NA	NA	NA	NA
28	present	NA	present	present	present	0.9	2.94	high	normal	NA	NA
29	present	NA	present	present	NA	NA	21.94	NA	decreased	normal	negative
30	present	NA	present	present	NA	NA	NA	NA	decreased	NA	negative
	present	NA	present	present	NA	NA	NA	NA	decreased	NA	negative
31	abscent	present	present	present	abscent	6.4	0.80	NA	normal	decreased	negative
32	present	present	present	present	abscent	4.6	3.96	NA	decreased	NA	NA
33	present	present	present	present	present	5.2	5.30	high	decreased	decreased	negative
34	abscent	present	present	present	NA	16.0	2.15	normal	decreased	NA	negative
35	present	present	present	present	present	3.2	5.58	NA	decreased	normal	negative
36	present	present	present	present	abscent	4.2	6.80	normal	decreased	decreased	negative
37	abscent	abscent	present	present	abscent	0.1	0.68	NA	decreased	decreased	postitive
	abscent	abscent	present	present	abscent	0.2	0.92	NA	decreased	decreased	postitive
38	present	NA	present	present	abscent	NA	5.20	NA	decreased	decreased	negative
39	present	NA	NA	NA	present	NA	5.90	NA	NA	NA	negative
40	present	present	present	present	abscent	6.6	4.70	NA	decreased	NA	negative
41	present	abscent	present	present	abscent	1.5	1.23	normal	decreased	decreased	negative
42	present	present	present	present	abscent	2.2	2.71	NA	decreased	normal	negative
43	present	present	present	present	NA	NA	9.20	NA	normal	normal	negative
44	present	abscent	present	present	present	2.0	2.79	high	normal	normal	negative
45	present	present	present	present	present	11.6	1.13	normal	normal	normal	negative
46	present	NA	present	present	abscent	NA	5.30	NA	normal	normal	negative
47	abscent	present	present	abscent	abscent	9.6	0.38	normal	normal	normal	NA
48	present	abscent	present	present	abscent	2.9	2.80	high	decreased	normal	negative
49	present	present	present	present	abscent	13.0	3.60	NA	normal	normal	negative
	present	present	present	present	NA	3.6	3.23	NA	normal	normal	negative
50	present	abscent	present	present	NA	2.9	7.40	NA	NA	NA	NA
51	present	abscent	present	present	abscent	NA	8.40	high	normal	NA	negative
52	present	abscent	present	present	abscent	NA	6.10	NA	normal	normal	negative
53	present	present	present	present	present	15.0	1.56	normal	normal	normal	negative
54	present	present	present	present	abscent	11.1	7.05	normal	decreased	decreased	negatuve
55	present	abscent	present	present	abscent	1.2	4.50	NA	decreased	normal	negative
56	abscent	abscent	present	present	NA	5.9	0.90	NA	decreased	normal	negative
57	present	present	present	present	present	11.0	1.50	normal	normal	normal	NA
58	present	present	present	present	abscent	4.6	2.23	NA	decreased	decreased	negative
59	present	abscent	present	present	abscent	3.1	4.10	NA	decreased	NA	NA
60	present	abscent	present	present	present	NA	1.30	high	decreased	decreased	NA
61	abscent	abscent	present	present	abscent	0.3	0.60	high	decreased	decreased	NA
62	present	abscent	present	present	NA	0.7	5.40	high	normal	normal	negative
63	present	abscent	present	present	abscent	NA	6.00	NA	decreased	decreased	NA
64	abscent	present	present	present	present	6.8	0.90	NA	normal	normal	NA
65	present	present	present	present	abscent	5.8	1.20	high	normal	normal	negative
66	present	present	present	present	present	10.4	4.90	normal	normal	normal	negative
67	abscent	abscent	present	present	abscent	0.6	NA	NA	NA	NA	NA
68	present	abscent	present	present	abscent	NA	9.20	NA	normal	normal	negative
69	NA	present	present	present	abscent	8.3	NA	NA	decreased	decreased	NA
70	present	present	present	present	abscent	6.2	3.10	NA	normal	normal	negative
71	present	abscent	present	present	abscent	1.1	5.80	high	normal	normal	negative
72	present	NA	NA	NA	abscent	NA	NA	NA	normal	normal	negative
73	present	NA	NA	present	NA	NA	10.40	NA	normal	normal	negative
74	present	abscent	present	present	abscent	2.5	3.20	high	normal	normal	negative
75	present	NA	present	present	NA	1.0	6.18	NA	decreased	decreased	NA

NA, not available

Ref. No.	Light microscopy		Immunofluorescence microscopy			Electron microscopy				
	main hstological diagnosis	Presence of crescents	IgG	IgA	C3	subepithelial EDD	subendothelial EDD	intramembranous EDD	mesangial EDD	hump
14	NA	NA	NA	present	NA	NA	NA	NA	NA	NA
15	endo + mesangial	present	NA	present	present	NA	NA	NA	NA	NA
16	endo + mesangial	NA	NA	present	NA	NA	NA	NA	NA	NA
17	endo	NA	present	present	present	abscent	present	abscent	present	abscent
18	endo	present	present	present	present	abscent	present	abscent	present	abscent
19	NA	NA	NA	NA	NA	NA	NA	NA	NA	NA
20	NA	NA	NA	NA	NA	NA	NA	NA	NA	NA
	endo	NA	present	present	present	NA	NA	NA	NA	NA
21	endo	NA	NA	present	present	NA	NA	NA	present	NA
22	endo	abscent	abscent	present	present	NA	NA	NA	NA	NA
23	endo	abscent	NA	present	present	NA	NA	NA	NA	NA
24	endo	present	abscent	present	present	abscent	present	abscent	present	abscent
25	endo + mesangial	present	NA	present	present	present	present	abscent	present	present
26	MPGN	abscent	abscent	abscent	abscent	abscent	present	abscent	abscent	abscent
27	mesangial	present	abscent	present	present	present	abscent	abscent	present	present
28	NA	NA	NA	NA	NA	NA	NA	NA	NA	NA
29	mesangial	present	NA	present	present	NA	NA	NA	NA	NA
30	endo	present	present	NA	present	NA	NA	NA	NA	NA
	endo	present	NA	present	present	NA	NA	NA	NA	NA
31	endo + mesangial	NA	abscent	present	present	NA	present	NA	NA	NA
32	endo + mesangial	NA	NA	present	present	NA	NA	NA	NA	NA
33	endo	NA	NA	present	present	NA	NA	NA	NA	NA
34	mesangial	present	abscent	present	present	present	NA	NA	NA	present
35	endo + mesangial	abscent	NA	present	present	present	NA	NA	NA	present
36	mesangial	present	NA	present	present	abscent	NA	NA	present	abscent
37	MPGN	present	NA	NA	present	NA	NA	NA	present	NA
	MPGN	NA	NA	NA	present	present	present	present	NA	NA
38	endo	present	present	present	present	NA	NA	NA	NA	NA
39	endo + mesangial	NA	abscent	present	present	present	present	NA	NA	present
40	endo + mesangial	NA	NA	present	present	present	present	NA	NA	present
41	MPGN	NA	NA	NA	present	NA	NA	NA	NA	NA
42	endo	present	present	present	present	present	present	NA	present	present
43	endo + mesangial	present	NA	present	present	present	NA	NA	NA	present
44	endo	present	abscent	present	present	present	NA	NA	NA	present
45	endo + mesangial	present	abscent	present	present	present	NA	NA	present	NA
46	endo + mesangial	NA	NA	present	NA	present	NA	NA	present	present
47	NA	NA	NA	NA	NA	NA	NA	NA	NA	NA
48	mesangial	present	present	present	present	abscent	abscent	abscent	present	abscent
49	endo + mesangial	present	abscent	present	present	abscent	present	NA	present	abscent
	endo + mesangial	NA	abscent	present	present	abscent	NA	NA	present	abscent
50	endo + mesangial	present	abscent	present	present	present	NA	NA	present	present
51	mesangial	NA	abscent	present	present	NA	NA	NA	NA	NA
52	crescentic	present	NA	present	present	abscent	abscent	abscent	present	abscent
53	endo	abscent	present	present	present	NA	present	NA	present	NA
54	crescentic	present	present	abscent	present	NA	NA	NA	NA	NA
55	endo + mesangial	NA	present	NA	present	NA	present	NA	present	NA
56	NA	NA	NA	NA	NA	NA	NA	NA	NA	NA
57	endo + mesangial	present	NA	present	present	NA	NA	NA	NA	NA
58	NA	NA	NA	NA	NA	NA	NA	NA	NA	NA
59	endo + mesangial	abscent	present	present	present	abscent	present	NA	present	abscent
60	MPGN & Mesangial	abscent	present	present	present	NA	NA	NA	NA	NA
61	MPGN	NA	NA	NA	present	NA	NA	NA	NA	NA
62	endo + mesangial	present	present	present	present	abscent	present	NA	present	abscent
63	crescentic	present	present	abscent	present	NA	NA	present	NA	NA
64	mesangial	abscent	present	present	present	NA	present	NA	present	NA
65	endo	present	present	present	present	NA	NA	NA	NA	NA
66	endo	present	NA	present	abscent	NA	NA	NA	NA	NA
67	mesangial	NA	present	present	present	NA	NA	NA	present	NA
68	crescentic	present	abscent	abscent	abscent	NA	NA	NA	NA	NA
69	MPGN	NA	NA	NA	NA	NA	NA	NA	NA	NA
70	MPGN	present	abscent	abscent	present	NA	present	NA	present	NA
71	mesangial	present	abscent	abscent	abscent	NA	NA	NA	NA	NA
72	endo + mesangial	NA	present	present	present	NA	NA	NA	NA	NA
73	endo + mesangial	NA	present	present	present	NA	present	NA	present	NA
74	DM + cresc	present	abscent	abscent	abscent	NA	NA	NA	NA	NA
75	endo	abscent	present	abscent	present	NA	NA	NA	NA	NA

NA, not available; endo, endothelial proliferative glomerulonephritis; mesangial, mesangial proliferative glomerulonephritis; MPGN, membranoproliferative glomerulonephritis; EDD, electron dense deposit

Ref. No.	treated with steroid	Outcome		
		death	ESKD	remission
14	no	death	HD	no
15	yes	alive	no	no
16	yes	death	no	no
17	yes	alive	no	yes
18	yes	alive	HD	no
19	no	alive	no	no
20	yes	alive	no	yes
	yes	alive	no	yes
21	yes	alive	no	yes
22	no	alive	no	no
23	yes	alive	HD	no
24	yes	alive	PD	no
25	yes	alive	no	yes
26	no	alive	no	yes
27	yes	alive	no	yes
28	yes	alive	no	no
29	yes	alive	HD	no
30	yes	alive	HD	no
	yes	alive	HD	no
31	no	alive	no	yes
32	no	alive	no	no
33	yes	alive	HD	no
34	yes	alive	HD	no
35	no	alive	no	yes
36	no	alive	HD	no
37	no	alive	no	yes
	yes	alive	no	yes
38	no	alive	no	yes
39	no	alive	HD	no
40	no	alive	no	no
41	NA	NA	NA	NA
42	yes	alive	no	yes
43	no	death	HD	no
44	yes	alive	no	yes
45	yes	alive	no	yes
46	no	alive	no	yes
47	no	alive	no	no
48	yes	alive	no	no
49	yes	alive	HD	no
	no	alive	no	no
50	yes	alive	no	no
51	no	alive	no	yes
52	no	alive	no	yes
53	yes	alive	no	no
54	no	alive	HD	no
55	no	alive	no	yes
56	no	alive	no	no
57	yes	alive	no	yes
58	no	alive	no	yes
59	no	alive	no	no
60	no	alive	no	no
61	no	alive	no	yes
62	yes	alive	no	yes
63	no	alive	no	no
64	no	alive	no	yes
65	yes	alive	no	yes
66	yes	death	HD	no
67	yes	alive	no	yes
68	no	alive	no	yes
69	no	alive	no	yes
70	no	alive	HD	no
71	yes	alive	HD	no
72	yes	death	HD	no
73	yes	alive	no	no
74	yes	death	no	no
75	no	alive	no	no

NA, not available; ESKD, end-stage kidney disease; HD, hemodialysis
