# Supplementary material for: Staphylococcus aureus Infection-Related Glomerulonephritis with Dominant IgA Deposition
Source: Int J Mol Sci. 2022 Jul 5;23(13):7482. doi: 10.3390/ijms23137482 (PMC9267153; doi:10.3390/ijms23137482)
Supplement: Supplementary file 1 [file ijms-23-07482-s001.zip › Table S2.pdf]

Table S2. Cohort studies of IgA-IRGN or SAGN.

Ref. No.	authors	year	category		No. of patients	age	male gender	underlying disease		Causative bacteria	
			IgA-IRGN	SAGN		mean ± SD (range)		diabetes	Cancer	Staphylo coccus	S. aureus
76	Ramineni S, et al.	2021	No	No	27	42.6 ± 15.1	20 (74%)	10 (37%)	NA	5 (19%)	NA
77	Ai S, et al.	2021	No	No	20	40.8 ± 13.0 (19-58)	16 (80%)	NA	NA	0 (0%)	0 (0%)
78	Grosser DS, et al.	2021	Yes	No	9	11.4 ± 3.3 (6-16)	8 (89%)	0 (0%)	0 (0%)	2 (22%)	1 (11%)
79	Huang Z, et al.	2021	Yes	No	50	42.8 ± 16.1 (19-80)	21 (42%)	4 (8%)	0 (0%)	2 (4%)	2 (4%)
80	Zhang M, et al.	2021	Yes	No	12	58.7 ± 21.2 (25-90)	10 (83%)	4 (33%)	NA	2 (17%)	NA
81	Miquelestorena-Standley E, et al.	2020	Yes	No	27	62 ± 15 (5-83)	23 (85%)	18 (69%)	2 (7%)	21 (78%)	20 (74%)
82	Kitamura M, et al.	2019	Yes	Yes	7	58.3 ± 21.1 (18-79)	7 (100%)	2 (29%)	NA	7 (100%)	7 (100%)
83	Sakthirajan R, et al.	2018	No	No	47	42 ± 14	29 (62%)	9 (19%)	NA	11 (23%)	11 (23%)
84	Handa T, et al.	2018	Yes	No	13	51 ± 17	10 (77%)	2 (15%)	NA	5 (38%)	5 (38%)
85	Khalighi MA, et al.	2018	Yes	Yes	5	58.8 ± 15.5 (33-75)	3 (60%)	NA	NA	5 (100%)	5 (100%)
86	Hemminger J, et al.	2018	Yes	No	9	54.0 ± 8.3 (43-68)	8 (89%)	2 (22%)	0 (0%)	2 (22%)	2 (22%)
87	Ramanathan G, et al.	2017	No	No	43	44 (34-53)	22 (51%)	26 (61%)	0 (0%)	9 (21%)	NA
88	Satoskar AA, et al.	2017	No	Yes	78	55.0 ± 12.1 (21-91)	61 (78%)	32 (41%)	NA	78 (100%)	59 (76%)
89	Dhanapriya J, et al.	2017	Yes	No	12	52.4 ± 13.0 (24-65)	8 (67%)	2 (17%)	0 (0%)	6 (50%)	6 (50%)
90	Boils CL, et al.	2015	No	No	49	48 (3-84)	38 (78%)	9 (18%)	1 (2%)	25 (51%)	23 (47%)
91	Murakami CA, et al.	2014	No	No	72	48 (41-53)	46 (64%)	17 (24%)	NA	25 (75%)	NA
92	Hamouda M, et al.	2014	No	No	50	36.8 ± 10.0 (30-80)	33 (66%)	5 (10%)	NA	6 (12%)	NA
93	Cossey LN, et al.	2014	Yes	No	19	NA	NA	19 (100%)	NA	NA	NA
94	Satoskar AA, et al.	2013	Yes	Yes	8	59.8 ± 15.8 (44-90)	7 (88%)	0 (0%)	2 (25%)	8 (100%)	7 (88%)
95	Koo TY, et al.	2012	Yes	No	7	64.7 ± 21.7 (31-84)	6 (86%)	1 (14%)	0 (0%)	2 (29%)	2 (29%)
96	Worawichawong S, et al.	2011	Yes	No	7	66.4 ± 15.5 (46-86)	5 (71%)	1 (14%)	0 (0%)	4 (57%)	4 (57%)
97	Wen YK, et al.	2011	Yes	No	10	57 ± 17 (23-74)	9 (90%)	1 (10%)	1 (10%)	3 (30%)	3 (30%)
			No	No	32	54 ± 19	20 (63%)	NA	NA	10/26 (38%)	NA
98	Nasr SH, et al.	2011	No	No	109	> 65	80 (73%)	53 (49%)	15 (14%)	50 (46%)	45 (41%)
			No	No	57	16-64	33 (58%)	13 (23%)	1 (2%)	8 (14%)	NA
99	Wen YK	2010	No	No	20	60.9 ± 14.7 (30-80)	14 (70%)	NA	NA	12 (60%)	12 (60%)
100	Wen YK, et al.	2010	No	No	21	60.4 ± 14.5 (30-80)	15 (71%)	NA	NA	12 (57%)	12 (57%)
101	Wen YK, et al.	2010	Yes	No	12	62.3 ± 16.9 (23-80)	9 (75%)	1 (8%)	NA	7 (58%)	6 (50%)
102	Hsieh YP, et al.	2010	No	No	21	60.4 ± 14.5 (30-80)	15 (71%)	NA	NA	12 (57%)	12 (57%)
103	Wen YK	2009	No	No	20	60.9 ± 14.7 (30-80)	14 (70%)	NA	NA	12 (60%)	12 (60%)
104	Haas M, et al.	2008	Yes	No	13	50 ± 22 (16-85)	11 (85%)	5 (38%)	NA	6 (46%)	6 (46%)
105	Zeledon JI, et al.	2008	No	No	4	59.5 ± 8.3 (53-71)	4 (100%)	0 (0%)	1 (25%)	2 (50%)	2 (50%)
106	Nasr SH, et al.	2008	No	No	86	56 ± 16	57 (66%)	25 (29%)	4 (5%)	21 (24%)	NA
107	Satoskar AA, et al.	2006	Yes	Yes	8	67.0 ± 7.8 (56-80)	6 (75%)	2 (25%)	1 (13%)	8 (100%)	7 (88%)
108	Nasr SH, et al.	2003	Yes	Yes	5	64.8 ± 15.0 (50-89)	4 (80%)	5 (100%)	NA	5 (100%)	3 (60%)
109	Nakamura T, et al.	2003	Yes	Yes	20	63.7 ± 10.2	12 (60%)	NA	NA	20 (100%)	20 (100%)
110	Nagaba Y, et al.	2002	Yes	Yes	8	53.0 ± 18.4 (23-75)	7 (88%)	NA	NA	8 (100%)	8 (100%)
111	Majumdar A, et al.	2000	No	No	62	57 (15-85)	39 (63%)	NA	NA	21/50 (42%)	17/50 (34%)
111	Majumdar A, et al.	2000	No	No	20	58.1± 10.1	14 (70%)	NA	NA	8 (40%)	6 (30%)
9-13	our data		Yes	Yes	28	61.3 ± 15.1	25 (89%)	NA	NA	28 (100%)	28 (100%)

IgA-IRGN, IgA-dominant deposition infection-related glomerulonephritis; SAGN, Staphylococcus infection-associated glomerulonephritis; NA, not available.

Ref. No.	Causative bacteria					Infection sites						
	S. epidermidis	other Staphylo coccus	Strepto coccus	others	unknown / not detected	skin infection	bone or joint	endocarditis	respiratory	visceral abscess	others	unknown
76	NA	NA	5 (19%)	5 (19%)	12 (44%)	5 (19%)	0 (0%)	0 (0%)	9 (33%)	0 (0%)	3 (11%)	10 (37%)
77	0 (0%)	0 (0%)	10 (50%)	4 (20%)	6 (30%)	0 (0%)	0 (0%)	20 (100%)	0 (0%)	0 (0%)	0 (0%)	0 (0%)
78	0 (0%)	1 (11%)	5 (56%)	1 (11%)	1 (11%)	0 (0%)	0 (0%)	0 (0%)	2 (22%)	0 (0%)	2 (22%)	5 (56%)
79	0 (0%)	0 (0%)	1 (2%)	7 (14%)	40 (80%)	4 (8%)	0 (0%)	0 (0%)	13 (26%)	0 (0%)	14 (28%)	19 (38%)
80	NA	NA	1 (8%)	1 (8%)	8 (67%)	3 (25%)	0 (0%)	1 (8%)	4 (33%)	0 (0%)	2 (17%)	2 (17%)
81	0 (0%)	1 (4%)	1 (4%)	8 (30%)	3 (11%)	11 (41%)	12 (44%)	4 (15%)	4 (15%)	0 (0%)	8 (30%)	3 (11%)
82	NA	NA	NA	NA	NA	NA	NA	NA	NA	NA	NA	NA
83	0 (0%)	0 (0%)	3 (6%)	11 (23%)	22 (47%)	18 (38%)	1 (2%)	0 (0%)	3 (6%)	0 (0%)	9 (19%)	16 (34%)
84	0 (0%)	0 (0%)	3 (23%)	0 (0%)	5 (38%)	5 (38%)	0 (0%)	2 (23%)	5 (38%)	0 (0%)	0 (0%)	1 (8%)
85	0 (0%)	0 (0%)	0 (0%)	0 (0%)	0 (0%)	3 (60%)	2 (40%)	0 (0%)	0 (0%)	0 (0%)	0 (0%)	0 (0%)
86	0 (0%)	0 (0%)	0 (0%)	3 (33%)	4 (44%)	3 (33%)	0 (0%)	0 (0%)	1 (11%)	0 (0%)	2 (22%)	3 (33%)
87	NA	NA	9 (21%)	6 (14%)	19 (44%)	20 (47%)	0 (0%)	0 (0%)	16 (37%)	0 (0%)	6 (14%)	1 (2%)
88	5 (6%)	7 (9%)	0 (0%)	7 (9%)	0 (0%)	17 (22%)	17 (22%)	18 (23%)	6 (8%)	6 (8%)	14 (18%)	0 (0%)
89	0 (0%)	0 (0%)	0 (0%)	4 (33%)	2 (17%)	6 (50%)	0 (0%)	0 (0%)	0 (0%)	0 (0%)	4 (33%)	2 (17%)
90	0 (0%)	2 (4%)	11 (22%)	8 (16%)	0 (0%)	0 (0%)	0 (0%)	49 (100%)	0 (0%)	0 (0%)	0 (0%)	0 (0%)
91	NA	NA	15 (21%)	11 (15%)	21 (29%)	8 (11%)	3 (4%)	16 (22%)	11 (15%)	0 (0%)	3 (4%)	30 (42%)
92	NA	NA	33 (66%)	NA	NA	17 (34%)	0 (0%)	0 (0%)	30 (60%)	0 (0%)	0 (0%)	5 (10%)
93	NA	NA	NA	NA	NA	NA	NA	NA	NA	NA	NA	NA
94	1 (13%)	0 (0%)	0 (0%)	0 (0%)	0 (0%)	4 (50%)	1 (13%)	1 (13%)	0 (0%)	2 (25%)	0 (0%)	0 (0%)
95	0 (0%)	0 (0%)	0 (0%)	1 (14%)	4 (57%)	NA	NA	NA	NA	NA	NA	NA
96	0 (0%)	3 (43%)	0 (0%)	0 (0%)	0 (0%)	3 (43%)	0 (0%)	1 (14%)	2 (29%)	0 (0%)	1 (14%)	0 (0%)
97	0 (0%)	0 (0%)	2 (20%)	5 (50%)	0 (0%)	1 (10%)	4 (40%)	0 (0%)	2 (20%)	1 (10%)	2 (20%)	0 (0%)
	NA	NA	NA	NA	NA	NA	NA	NA	NA	NA	NA	NA
98	2 (2%)	3 (3%)	17 (16%)	13 (12%)	37 (34%)	31 (28%)	8 (7%)	7 (6%)	30 (28%)	5 (5%)	20 (18%)	19 (17%)
	NA	NA	19 (33%)	NA	NA	6 (11%)	NA	NA	27 (48%)	NA	NA	NA
99	0 (0%)	0 (0%)	3 (15%)	4 (20%)	1 (5%)	2 (10%)	3 (15%)	4 (20%)	3 (15%)	2 (10%)	5 (40%)	1 (5%)
100	NA	NA	NA	NA	NA	5 (24%)	3 (14%)	4 (19%)	4 (19%)	2 (10%)	3 (14%)	0 (0%)
101	1 (8%)	0 (0%)	2 (17%)	3 (25%)	0 (0%)	NA	NA	NA	NA	NA	NA	NA
102	NA	NA	NA	NA	NA	5 (24%)	3 (14%)	4 (19%)	4 (19%)	2 (10%)	3 (14%)	0 (0%)
103	0 (0%)	0 (0%)	3 (15%)	4 (20%)	1 (5%)	2 (10%)	3 (15%)	4 (20%)	3 (15%)	2 (10%)	5 (40%)	1 (5%)
104	0 (0%)	0 (0%)	0 (0%)	3 (23%)	4 (31%)	3 (23%)	0 (0%)	0 (0%)	1 (8%)	1 (8%)	4 (31%)	4 (31%)
105	0 (0%)	0 (0%)	2 (50%)	0 (0%)	0 (0%)	0 (0%)	1 (25%)	2 (50%)	0 (0%)	0 (0%)	1 (25%)	0 (0%)
106	NA	NA	24 (28%)	5 (6%)	36 (42%)	16 (19%)	4 (5%)	10 (12%)	35 (41%)	2 (2%)	5 (6%)	14 (16%)
107	1 (13%)	0 (0%)	0 (0%)	0 (0%)	0 (0%)	4 (50%)	0 (0%)	1 (13%)	0 (0%)	1 (13%)	2 (25%)	0 (0%)
108	2 (40%)	0 (0%)	1 (20%)	0 (0%)	0 (0%)	4 (80%)	1 (20%)	0 (0%)	0 (0%)	1 (20%)	0 (0%)	0 (0%)
109	0 (0%)	0 (0%)	0 (0%)	0 (0%)	0 (0%)	NA	NA	NA	NA	NA	NA	NA
110	0 (0%)	0 (0%)	0 (0%)	0 (0%)	0 (0%)	3 (38%)	0 (0%)	0 (0%)	2 (25%)	1 (13%)	2 (25%)	0 (0%)
111	4/50 (8%)	0 (0%)	17/50 (34%)	6/50 (12%)	6/50 (12%)	0 (0%)	0 (0%)	62 (100%)	0 (0%)	0 (0%)	0 (0%)	0 (0%)
111	2 (10%)	0 (0%)	3 (15%)	2 (10%)	7 (35%)	0 (0%)	0 (0%)	20 (100%)	0 (0%)	0 (0%)	0 (0%)	0 (0%)
9-13	0 (0%)	0 (0%)	0 (0%)	0 (0%)	0 (0%)	3 (11%)	1 (4%)	0 (0%)	8 (29%)	14 (50%)	2 (7%)	0 (0%)

Ref. No.	symptomes					laboratory data					
	AKI or RPGN	nephrotic syndrome	proteinuria	hematuria	purpura	proteinuria (g/day)	serum creatinine (mg/dL)	elevated serum IgA levels	decreased C3 levels	decreased C4 levels	positive test for ANCA
76	26 (96%)	1 (4%)	NA	NA	NA	4.1 ± 1.4	5.7 ± 2.47	NA	24 (89%)	NA	NA
77	12 (60%)	NA	19 (95%)	20 (100%)	20 (100%)	2.64 ± 2.08 (0.0-5.8)	5.95 ± 2.99 (1.3-11.3)	NA	13/17 (76%)		7/17 (41%)
78	7 (78%)	4 (44%)	9 (100%)	9 (100%)	NA	NA	1.92 ± 0.93 (0.8-3.4)	NA	NA	NA	NA
79	NA	NA	NA	NA	NA	3.24 ± 3.41	1.39 ± 1.07	NA	NA	NA	NA
80	10 (83%)	7 (58%)	12 (100%)	12 (100%)	2 (17%)	5.14 ± 3.05 (0.7-10.3)	3.28 ± 2.11 (0.9-8.3)	NA	8 (67%)		0/11 (0%)
81	15 (57%)	18 (67%)	NA	20/21 (95%)	NA	5.0 ± 3.4 (0.4-16.4)	4.24 ± 2.93	11/13 (85%)	4/25 (16%)	2/26 (8%)	4/15 (27%)
82	NA	NA	6 (86%)	7 (100%)	NA	4.78 ± 3.75 (0.1-10.1)	2.45 ± 1.59 (0.5-4.8)	NA	0/6 (0%)	0/6 (0%)	NA
83	37 (79%)	26 (55%)	47 (100%)	40 (85%)	NA	NA	NA	NA	47 (100%)	4 (9%)	NA
84	NA	NA	NA	11 (85%)	NA	5.3 ± 6.4 (0.01-19.06)	1.49 ± 0.80 (0.57-3.19)	NA	4 (31%)	3 (23%)	NA
85	5 (100%)	NA	4/4 (100%)	4/4 (100%)	NA	NA	3.94 ± 1.54 (1.7-6.0)	5 (100%)	4/4 (100%)	0 (0%)	NA
86	9 (100%)	NA	9 (100%)	9 (100%)	1 (11%)	13.2 ± 13.2 (0.4-38)	4.82 ± 1.60 (2.7-7.7)	NA	5/8 (63%)		0 (0%)
87	NA	4 (9%)	NA	34 (79%)	NA	2.33 (1.31-4.79)	0.97 (0.81-1.27)	NA	38 (88%)	14 (33%)	8 (19%)
88	NA	35/73 (48%)	73/73 (100%)	NA	16 (21%)	NA	NA	NA	19/64 (30%)	9/64 (14%)	9/41 (22%)
89	11 (92%)	11 (92%)	12 (100%)	12 (100%)	NA	5.23 ± 1.85 (1.8-8.5)	5.65 ± 2.44 (1.5-8.7)	NA	1 (8%)	0 (0%)	NA
90	40/47 (85%)	3/47 (6%)	NA	36/37 (97%)	NA	1.8 (0.5-15)	3.8 (1.0-12.0)	NA	17/32 (53%)	6/32 (19%)	8/29 (28%)
91	NA	19 (26%)	NA	31 (43%)	NA	NA	2.5 (1.5-4.9)	NA	12 (17%)	12 (17%)	NA
92	NA	5 (10%)	NA	NA	NA	NA	2.14	NA	31/42 (74%)	8/42 (19%)	0/2 (0%)
93	16 (84%)	5 (26%)	NA	NA	NA	NA	NA	NA	NA	NA	NA
94	8 (100%)	NA	7 (88%)	8 (100%)	8 (100%)	NA (0-10)	3.83 ± 2.59 (1.9-9.7)	NA	2/7 (29%)	0/7 (0%)	NA
95	7 (100%)	NA	7 (100%)	7 (100%)	NA	5.34 ± 1.87 (3.2-8.9)	3.13 ± 1.02 (1.9-4.7)	5 (71%)	2 (29%)	2 (29%)	NA
96	6 (86%)	2 (29%)	7 (100%)	7 (100%)	NA	NA	8.09 ± 2.71	NA	1 (14%)	1 (14%)	NA
97	9 (90%)	6 (60%)	10 (100%)	10 (100%)	NA	3.9 ± 3.6 (0.1-9.8)	5.1 ± 3.8 (1.2-11.8)	6/8 (75%)	6 (60%)		NA
	NA	NA	NA	NA	NA	2.9 ± 2.9	6.5 ± 5.6	4/22 (18.2%)	24 (75%)		NA
98	NA	23/87 (26%)	72/72 (100%)	98/103 (95%)	NA	3.6	5.1	NA	57/83 (69%)	29/83 (35%)	5/66 (8%)
	NA	NA	NA	NA	NA	3.8	3.8	NA	NA	NA	NA
99	18 (90%)	8 (40%)	20 (100%)	20 (100%)	NA	3.2 ± 2.6 (0.5-9.8)	6.7 ± 3.3 (1.7-11.8)	NA	12 (60%)	NA	NA
100	21 (100%)	NA	NA	21 (100%)	NA	3.2 (0.5-9.8)	6.5 ± 3.3 (1.7-11.8)	NA	13 (62%)	NA	NA
101	10 (83%)	NA	NA	12 (100%)	NA	3.9 ± 3.2 (0.4-9.8)	5.0 ± 3.5 (0.6-11.8)	6/9 (67%)	7 (58%)		NA
102	21 (100%)	NA	NA	21 (100%)	NA	3.2 (0.5-9.8)	6.5 ± 3.3 (1.7-11.8)	NA	13 (62%)	NA	NA
103	18 (90%)	8 (40%)	20 (100%)	20 (100%)	NA	3.2 ± 2.6 (0.5-9.8)	6.7 ± 3.3 (1.7-11.8)	NA	12 (60%)	NA	NA
104	NA	6/10 (60%)	10/10 (100%)	13 (100%)	NA	4.8 ± 3.0 (1.6-11.0)	4.4 ± 2.7 (1.4-9.5)	NA	4/10 (40%)	NA	NA
105	4 (100%)	3 (75%)	4 (100%)	4 (100%)	NA	4.6 ± 3.7 (0.7-9.6)	8.0 ± 4.2 (2.7-11.8)	NA	3 (75%)	1 (25%)	1 (25%)
106	NA	25 (29%)	NA	77 (90%)	NA	3.7	4.36	NA	37/58 (64%)	28/58 (48%)	NA
107	NA	NA	8 (100%)	8 (100)	NA	6.4 ± 3.4 (2.5-9.0)	4.6 ± 2.4 (1.3-9.5)	NA	2 (25%)	1 (13%)	NA
108	5 (100%)	NA	3/3 (100%)	5 (100%)	NA	1.6 ± 1.2 (0.2-2.5)	8.0 ± 3.0 (4.1-10.4)	1/1 (100%)	5 (100%)	2 (40%)	NA
109	NA	NA	NA	NA	NA	2.6 ± 0.6	2.3 ± 1.2	NA	NA	NA	NA
110	5 (63%)	NA	8 (100)	8 (100)	7 (88%)	3.8 ± 2.7	3.6 ± 2.9	6 (75%)	2 (25%)	0 (0%)	0/2 (0%)
111	NA	NA	NA	NA	NA	NA	NA	NA	NA	NA	NA
111	NA	NA	NA	NA	NA	NA	5.88± 3.03 (1.9-12.2)	NA	NA	NA	NA
9-13	14	18	28	20	6	5.7 ± 4.6	2.9 ± 2.4	24 (86%)	1 (4%)	0 (0%)	0 (0%)

Ref. No.	Light microscopy					Immunofluorescence microscopy			Electron microscopy				
	mesangial GN	endocapillary GN	MPGN	necrotizing / crescenic	presence of crescents	IgG	IgA	C3	subepi. EDD	subendo. EDD	intramemb. EDD	mesangial EDD	hump
76	1 (4%)	24 (4%)	1 (89%)	5 (19%)	19 (70%)	19 (70%)	1 (4%)	27 (100%)	NA	NA	NA	NA	NA
77	5/5 (100%)	2/5 (40%)	0 (0%)	1/5 (20%)	4/5 (80%)	2/5 (40%)	3/5 (60%)	4/5 (80%)	0/2 (0%)	1/2 (50%)	0/2 (0%)	1/2 (50%)	0/2 (0%)
78	9 (100%)	8 (89%)	0 (0%)	0 (0%)	5 (56%)	7 (78%)	9 (100%)	9 (100%)	6 (67%)	8 (89%)	1 (11%)	9 (100%)	4 (44%)
79	47 (94%)	40 (80%)	3 (6%)	1 (2%)	38 (76%)	14 (28%)	50 (100%)	50 (100%)	3/14 (21%)	1/14 (7%)	0 (0%)	10/14 (71%)	1/14 (7%)
80	2 (17%)	10 (83%)	0 (0%)	0 (0%)	9 (75%)	5 (42%)	12 (100%)	12 (100%)	5/10 (50%)	6/10 (60%)	NA	10/10 (100%)	5/10 (50%)
81	24 (89%)	24 (89%)	9 (33%)	NA	10 (37%)	4 (15%)	27 (100%)	27 (100%)	13 (48%)	NA	3 (11%)	NA	13 (48%)
82	3 (43%)	5 (71%)	0 (0%)	1 (13%)	4 (57%)	NA	7 (100%)	6 (86%)	3/6 (50%)	3/6 (50%)	NA	6/6 (100%)	NA
83	NA	NA	NA	47 (100%)	47 (100%)	33 (70%)	7 (15%)	33 (70%)	NA	NA	NA	NA	NA
84	10 (77%)	11 (85%)	NA	NA	NA	4 (31%)	13 (100%)	11 (85%)	4 (31%)	5 (38%)	1 (8%)	6 (46%)	3 (23%)
85	5 (100%)	5 (100%)	1 (20%)	0 (0%)	4 (80%)	3 (60%)	5 (100%)	5 (100%)	2 (40%)	5 (100%)	2 (40%)	5 (100%)	1 (20%)
86	9 (100%)	8 (89%)	0 (0%)	0 (0%)	3 (33%)	9 (100%)	9 (100%)	9 (100%)	5 (56%)	9 (100%)	7 (78%)	9 (100%)	1 (11%)
87	2 (5%)	35 (81%)	2 (5%)	4 (9%)	27 (63%)	17/35 (49%)	10/35 (29%)	34/35 (97%)	31/38 (82%)	9/36 (25%)	NA	25/35 (71%)	31/38 (82%)
88	common	47 (60%)	NA	NA	27 (35%)	32 (41%)	58 (74%)	67 (86%)	24 (31%)	NA	NA	frequent	24 (31%)
89	2 (17%)	3 (25%)	0	7 (58%)	8 (67%)	1 (8%)	12 (100%)	12 (100%)	4/4 (100%)	0 (0%)	0 (0%)	0 (0%)	0 (0%)
90	5 (10%)	18 (37%)	0 (0%)	26 (53%)	33 (67%)	13 (27%)	14 (29%)	46 (94%)	17 (35%)	22 (45%)	0 (0%)	41 (84%)	7 (14%)
91	NA	NA	NA	NA	3 (4%)	15 (21%)	3 (4%)	45 (63%)	48 (67%)	26 (36%)	0 (0%)	49 (68%)	48 (67%)
92	2/16 (13%)	12/16 (75%)	NA	2/16 (13%)	2/16 (13%)	NA	NA	NA	NA	NA	NA	NA	NA
93	NA	NA	NA	NA	NA	2 (11%)	19 (100%)	15 (79%)	NA	NA	NA	NA	NA
94	7 (88%)	7 (88%)	0 (0%)	0 (0%)	3 (38%)	8 (100%)	8 (100%)	8 (100%)	5 (63%)	2 (25%)	0 (0%)	7 (88%)	2 (25%)
95	7 (100%)	7 (100%)	0 (0%)	1 (14%)	5 (71%)	6 (86%)	7 (100%)	7 (100%)	5/6 (83%)	1/6 (17%)	4/6 (67%)	6/6 (100%)	5/6 (83%)
96	7 (100%)	7 (100%)	1 (14%)	1 (14%)	1 (14%)	5 (71%)	7 (100%)	7 (100%)	7 (100%)	5 (71%)	3 (43%)	7 (100%)	7 (100%)
97	0 (0%)	10 (100%)	0 (0%)	3 (30%)	3 (30%)	8 (80%)	10 (100%)	10 (100%)	10 (100%)	3 (30%)	0 (0%)	9 (90%)	10 (100%)
	NA	NA	NA	NA	NA	NA	NA	NA	NA	NA	NA	NA	NA
98	14 (13%)	88 (81%)	1 (1%)	5 (5%)	40 (37%)	39/97 (40%)	38/97 (39%)	97/97 (100%)	100 (92%)	72 (66%)	0 (0%)	95 (87%)	100 (92%)
	NA	NA	NA	NA	NA	NA	NA	NA	NA	NA	NA	NA	NA
99	7 (35%)	13 (65%)	0 (0%)	4 (20%)	9 (45%)	14 (70%)	15 (75%)	20 (100%)	13 (65%)	5 (25%)	0 (0%)	12 (60%)	13 (65%)
100	8 (38%)	13 (62%)	0 (0%)	4 (19%)	9 (43%)	14 (67%)	9 (43%)	21 (100%)	9/12 (75%)	5/12 (42%)	0/12 (0%)	11/12 (92%)	9/12 (75%)
101	10 (83%)	4 (33%)	0	NA	4 (33%)	NA	12 (100%)	12 (100%)	5 (42%)	NA	NA	NA	4 (33%)
102	8 (38%)	13 (62%)	0 (0%)	4 (19%)	9 (43%)	14 (67%)	9 (43%)	21 (100%)	9/12 (75%)	5/12 (42%)	0/12 (0%)	11/12 (92%)	9/12 (75%)
103	7 (35%)	13 (65%)	0 (0%)	4 (20%)	9 (45%)	14 (70%)	15 (75%)	20 (100%)	13 (65%)	5 (25%)	0 (0%)	12 (60%)	13 (65%)
104	13 (100%)	6 (46%)	0 (0%)	0 (0%)	4 (31%)	5 (38%)	13 (100%)	13 (100%)	13 (65%)	6 (46%)	10 (77%)	13 (65%)	13 (65%)
105	2 (50%)	3 (75%)	0 (0%)	1 (25%)	2 (50%)	2 (50%)	2 (50%)	3 (75%)	2 (50%)	0 (0%)	0 (0%)	2 (50%)	2 (50%)
106	7 (8%)	73 (85%)	2 (2%)	4 (5%)	39 (45%)	55/84 (66%)	37/84 (44%)	84/84 (100%)	78/83 (94%)	62/83 (75%)	0 (0%)	75/83 (90%)	75/83 (90%)
107	6 (75%)	4 (50%)	0 (0%)	0 (0%)	0 (0%)	3 (38%)	8 (100%)	7 (88%)	6 (75%)	1 (13%)	0 (0%)	8 (100%)	0 (0%)
108	5 (100%)	5 (100%)	0 (0%)	0 (0%)	0 (0%)	4 (80%)	5 (100%)	5 (100%)	5 (100%)	4 (80%)	0 (0%)	5 (100%)	5 (100%)
109	NA	NA	NA	NA	NA	NA	NA	NA	NA	NA	NA	NA	NA
110	4 (50%)	0 (0%)	0 (0%)	4 (50%)	7 (88%)	1 (13%)	8 (100%)	8 (100%)	1 (13%)	1 (13%)	0 (0%)	8 (100%)	0 (0%)
111	NA	NA	NA	NA	NA	NA	NA	NA	NA	NA	NA	NA	NA
111	2 (10%)	NA	3 (15%)	NA	6 (30%)	NA	NA	NA	NA	NA	NA	NA	NA
9-13	12/17 (71%)	5/17 (29%)	1/17 (6%)	2/17 (12%)	13/17 (76%)	14/17 (82%)	17/17 (100%)	17/17 (100%)	5/17 (29%)	7/17 (41%)	0/17 (0%)	8/17 (47%)	5/17 (29%)
